# Effect of twin block on intracranial pressure

**DOI:** 10.1007/s00784-025-06214-7

**Published:** 2025-02-19

**Authors:** Yasin Hezenci, Musa Bulut, Oğuzhan Demirel

**Affiliations:** 1 Department of Orthodontics, Bolu Abant Izzet Baysal University, Bolu, Turkey; 2 Department of Dentomaxillofacial Radiology, Bolu Abant Izzet Baysal University, Bolu, Turkey

**Keywords:** Intracranial pressure, Optic nerve, Twin block

## Abstract

**Objectives:**

This study aimed to investigate the impact of Twin Block appliances on intracranial pressure (ICP) in adolescents by measuring the optic nerve sheath diameter (ONSD) using ultrasonography.

**Methods:**

We conducted a prospective study involving 20 adolescents (8 girls and 12 boys) with skeletal mandibular retrognathia undergoing treatment with Twin Block appliances. ONSD measurements were taken at six different time points: before appliance placement (T0), 1 min after placement (T1), 10 min after placement (T2), one month after the start of treatment with twin block (T3), immediately after appliance removal (T4), and 10 min after removal (T5). Mean arterial pressure (MAP), heart rate, and peripheral oxygen saturation (SpO2) were also monitored. Statistical analyses were performed using Friedman and Wilcoxon Signed-Rank tests with Bonferroni correction, considering *p* < 0.05 as statistically significant.

**Results:**

Significant increases in ONSD were observed at T1 and T2 compared to T0 (*p* < 0.05), with the highest ONSD recorded at T1. No significant changes in ONSD were noted at T3 or T5, indicating that the initial increase in ONSD was temporary. MAP showed a significant decrease at T2, but no significant changes were observed in SpO2 or heart rate across the time points.

**Conclusion:**

This study is the first to report a significant increase in ICP, as indicated by ONSD, in adolescents using Twin Block appliances. The observed rise in ONSD shortly after appliance placement suggests a temporary increase in ICP. These findings highlight the importance of monitoring intracranial pressure during orthodontic treatment, particularly in adolescents.

**Supplementary Information:**

The online version contains supplementary material available at 10.1007/s00784-025-06214-7.

## Introduction

Orthodontic malocclusions refer to misalignments of the teeth and jaws that can impact both oral function and appearance. These malocclusions are categorized into several classes based on their specific characteristics and severity. Class II malocclusions are one of the most common and therefore one of the most frequently treated problems by orthodontists. It mostly occurs due to posterior positioning of the mandible or developmental delay. Class II malocclusions can lead to a range of functional issues, including difficulty in biting and chewing, as well as aesthetic concerns.

Effective treatment of the Class II malocclusions often involves a combination of orthodontic appliances and, in some cases, orthopedic interventions to correct the malocclusion and improve both function and appearance. The ideal treatment approach for patients with Class II malocclusion characterized with mandibular retrognathia in the developmental period is to stimulate mandibular growth and development. For this purpose, functional appliances are often preferred [1]. Twin block appliances are removable functional appliances that are frequently preferred in orthodontic clinics due to their advantages such as being easily attached and removed by the patient, easy to maintain oral hygiene and low cost.

Numerous studies have explored the impact of orthopedic treatment forces on maxillofacial structures. Orthopedic effects resulting from orthodontic treatment involve alterations in the positioning of skull bones [2]. These changes are not confined to the area where the force is applied; rather, substantial orthopedic forces influence both the specific application site and the surrounding craniofacial structures.

The optic nerve, a cranial nerve encased in meninges, is surrounded by cerebrospinal fluid (CSF) within the subarachnoid space that envelops it [3]. Elevated intracranial pressure (ICP) can lead to an increase in CSF volume in this space, resulting in an expansion of the optic nerve sheath diameter (ONSD) [4]. Intracranial or intraventricular microsensor devices are considered the gold standard for measuring ICP [5, 6], though they carry risks of infections or hemorrhages. Helmke and Hansen [7] first introduced ultrasonographic examination of ONSD as a simple, accurate, safe, and noninvasive alternative for assessing ICP. Numerous studies have validated the reliability of this technique [4, 8–10], with Geeraerts et al. finding that an ONSD greater than 5.8 mm indicates an ICP exceeding 20 mmHg [9].

Previous studies have indicated that mouth gags used in surgical procedures such as adenoidectomy, which indirectly shift the tongue and mandible forward, can lead to an increase in intracranial pressure [11, 12]. However, there are limited studies examining the effects of orthodontic orthopedic appliances on intracranial pressure [13, 14]. In orthodontic practice, children and adolescents frequently use functional appliances, but those with medical conditions such as vasculitis, hydrocephalus, pseudotumor cerebri, or liver failure are at higher risk for elevated ICP.

Therefore, the aim of our study is to investigate the effect of the use of the twin block appliance on intracranial pressure by measuring the optic nerve sheath diameter ultrasonographically.

## Materials and methods

This prospective study was conducted in accordance with the Declaration of Helsinki and was approved by Bolu Abant Izzet Baysal University Clinical Researches Ethical Committee. Each patient joined the study voluntarily and each parent/legal guardian signed an informed consent form. 20 patients (8 girl/ 12 boy) with skeletal mandibular retrognathia who were to be treated with Twin Block appliance are selected. The inclusion criteria included an overjet greater than 5 mm, bilateral class II molar relationships, and an ANB angle greater than 4°. All participants were at the maximum pubertal growth spurt according to the methods described by Björk, Grave, and Brown [15, 16] for the hand-wrist maturation stage. Patients with a history of neurological conditions, previous eye surgeries, or eye diseases such as glaucoma, diabetic retinopathy, or papilloedema were excluded from the study.

The Twin Block appliance was made up of acrylic plates for both the upper and lower arches, designed to fit over the teeth and supporting structures. Adams and Ball clasps were incorporated into the upper molars and premolars to secure the upper portion, while ball clasps were placed on the mandibular anterior teeth for retention. While the degree of sagittal activation of the appliance varied based on the individual’s overjet, the average activation was approximately 7 mm. Initial measurements (T0) were taken before the appliance was placed in the patient’s mouth. After these measurements, Twin Block appliance was inserted, allowing the patient to position their mandible more anteriorly. The second set of measurements (T1) was recorded 1 min after the appliance was placed, followed by the third set of measurements (T2) 10 min later. Patients were then instructed to use the appliance regularly. The duration of appliance use was verified by the patient and their family. Patients who were found to not use it regularly were excluded from the study without undergoing the T3 measurement. One month after the start of appliance use, patients were scheduled for a follow-up visit where the fourth set of measurements (T3) was taken with the appliance in place. The appliance was then removed, and the fifth set of measurements (T4) was recorded. Finally, the sixth set of measurements (T5) was taken after a 10-minute wait. Thus, the changes in ONSD that occurred when the appliance was first used were examined. It was also examined whether there was a change in ONSD when the appliance was removed during routine appliance use. Mean arterial pressure (MAP), heart rate (BPM) and peripheral oxygen saturation levels (SpO_2_) were also measured at all times.

**Fig. 1:**
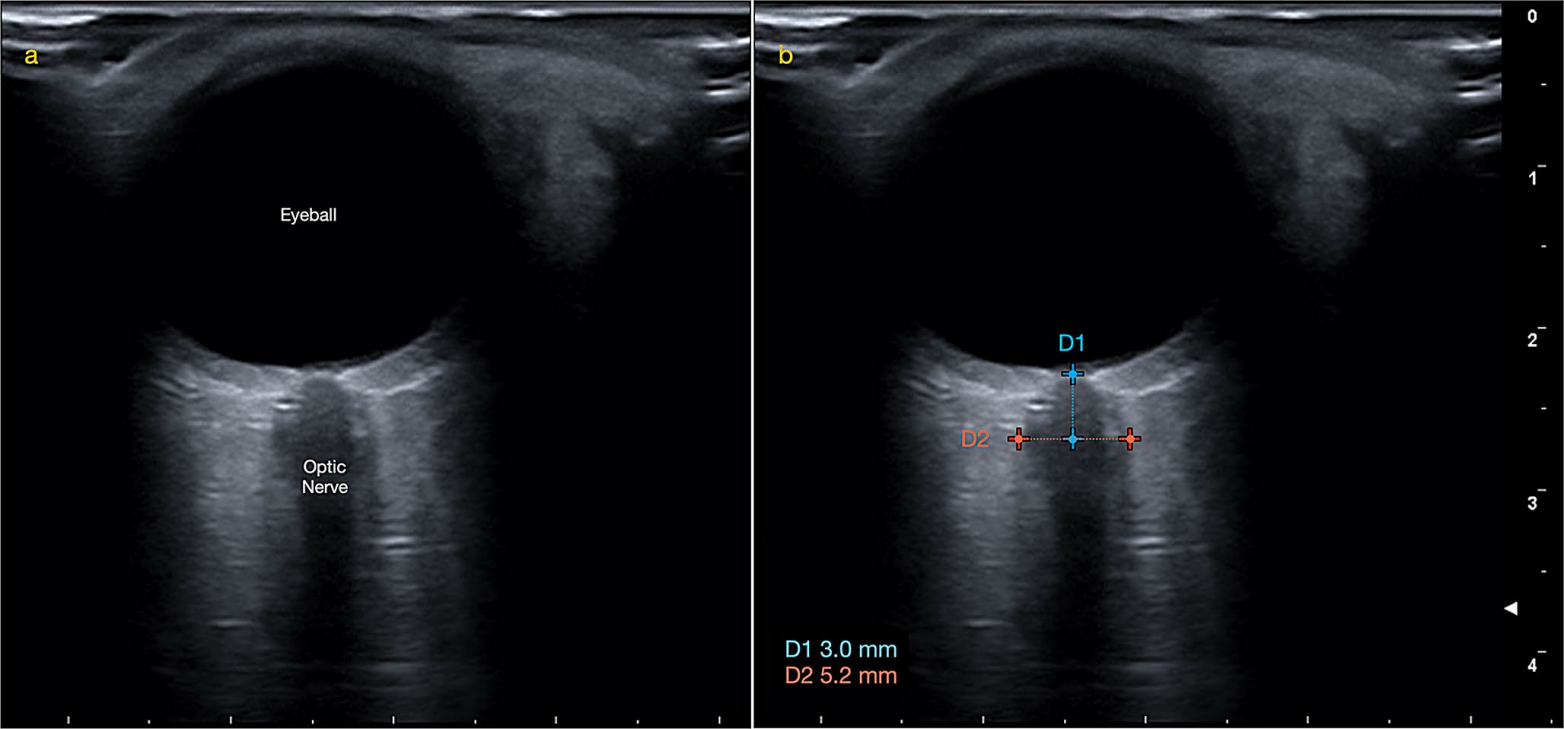
(**a**) The ultrasonographic image of the eyeball and optic nerve. (**b**) The measurement of the optic nerve sheath diameter (ONSD) was taken 3 mm behind the optic disc

The optic nerve sheath diameter was measured using a MyLab™X7 ultrasonography device (Esaote SPA, Genova, Italy) equipped with a high-frequency linear probe (> 7.5 MHz). An experienced Dentomaxillofacial radiologist (OD) conducted all ONSD measurements. Patients were examined in a seated position with their heads supported by the headrest of a dental unit and their eyes closed. Thick conductive ultrasound gel was applied to the eyeballs, and the probe was placed gently. The ultrasound settings were adjusted to achieve optimal resolution between the retrobulbar echogenic adipose tissue and the vertical hypoechoic band. ONSD measurements were taken 3 mm posterior to the optic disc for both eyes, and the average of these measurements was calculated (Fig. 1).

### Statistical analysis

The sample size was determined using the G*power 3.1.9.7 software (Heinrich-Heine-University, Düsseldorf, Germany), aiming to detect a difference of 1 mm and a power of 80%. The significance level for each analysis of variance was set at 0.003 to maintain the experiment-wide α level at 0.05. This calculation indicated that 12 patients were required [17]. To evaluate the changes in repeated measures (ONSD, SpO2, MAP, and heart rate), the Friedman test was applied. Intergroup differences were assessed using the Wilcoxon Signed-Rank test with Bonferroni correction, considering a p-value of < 0.05 as statistically significant. All statistical analyses were performed with SPSS v.26 (IBM, NY, USA).

## Results

**Fig. 2:**
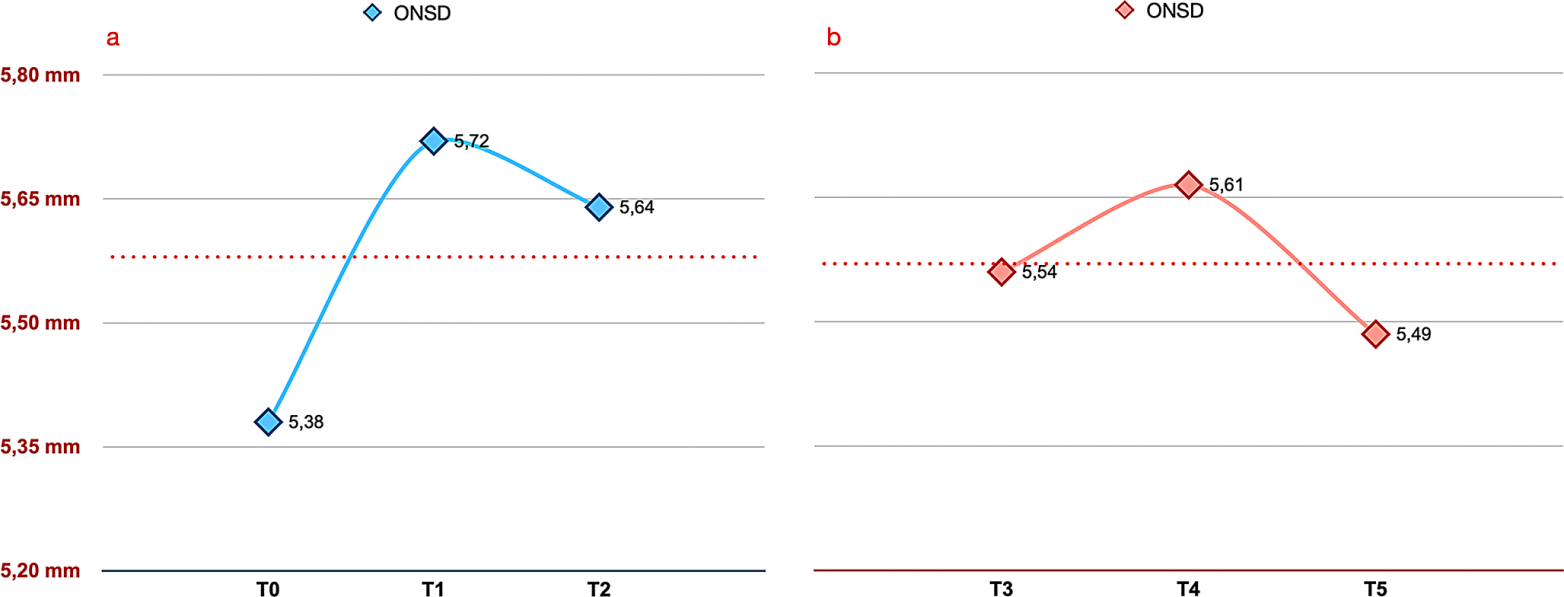
Optic nerve sheath diameter (ONSD) values expressed as mean. (**a**) T0: before Twin Block use, T1: 1 min after the appliance placed in situ T2: 10 min after the appliance placed in situ. (**b**) T3: 1 month after Twin Block use with the appliance still is in patient’s mouth, T4: 1 min after removing the appliance T5: 10 min after removing the appliance

The mean age for patients was 13.84 ± 1.46. The ONSD measurements taken at one minute (T1) and 10 min (T2) after the initial use of the Twin Block appliance were significantly higher than the baseline measurement taken before the appliance was used (T0) (*p* < 0.05). The highest ONSD values were measured at the first minute (T1) of appliance use (Fig. 2a; Table 1).

**Table 1:** Optic nerve sheath diameter, SpO2, heart rate and mean arterial pressure values associated with intracranial pressure at the start of Twin Block treatment

	T0	T1	T2	*p*	Difference
	Mean ± SD	Mean ± SD	Mean ± SD		
ONSD (mm)	5.38 ± 0.30	5.72 ± 0.26	5.64 ± 0.32	0.001^†*^ 0.001^*^ 0.001^*^ 0.368	T0-T1^‡^ T0-T2^‡^ T1-T2^‡^
Oxygen Saturation (%)	96.60 ± 2.37	95.50 ± 3.85	96.40 ± 2.30	0.696	-
Heart Rate (beats per minute)	85.10 ± 17.43	77.87 ± 21.98	83.30 ± 15.42	0.463	-
Mean Arterial Pressure (mmHg)	80.68 ± 14.36	75.98 ± 13.08	73.77 ± 9.62	0.027^†*^ 0.040^*^	T0-T2^‡^

† Friedman Test, ‡ Wilcoxon Signed-Rank Test, * p<0.05

No statistically significant difference was observed in the ONSD measurements taken after 1 month of Twin Block use (*p* > 0.05). Although it was observed that the ONSD value increased in the measurements made after the appliance was removed from the mouth (T4), no statistically significant difference was found (Fig. 2b; Table 2).

**Table 2:** Optic nerve sheath diameter, SpO2, heart rate and mean arterial pressure values associated with intracranial pressure after the one month of Twin Block use

	T3	T4	T5	*p*	Difference
	Mean ± SD	Mean ± SD	Mean ± SD		
ONSD (mm)	5.54 ± 0.37	5.61 ± 0.42	5.49 ± 0.43	0.130	-
Oxygen Saturation (%)	96.25 ± 2.69	96.00 ± 2.62	96.15 ± 2.18	0.824	-
Heart Rate (beats per minute)	83.80 ± 15.83	77.45 ± 27.72	83.65 ± 13.89	0.891	-
Mean Arterial Pressure (mmHg)	77.28 ± 11.12	74.55 ± 9.96	73.93 ± 11.42	0.078	-

† Friedman Test,* p< 0.05

No statistical significance was observed when the SpO2 and Heart Rate values of T0, T1, T2 were compared (Table 1). However, there was a statistically significant decrease in MAP after 10 min of Twin Block use. No statistical significance was observed when the SpO2, MAP and Heart Rate values of T3, T4, T5 were compared (Table 2).

No significant differences were found during measurements based on gender.

## Discussion

Our study aimed to evaluate the impact of Twin Block appliances on ICP by measuring ONSD ultrasonographically. We found that ONSD increased significantly within the first minute of appliance use (T1) and remained elevated at 10 min (T2) compared to baseline (T0). This indicates an immediate effect of the appliance on ONSD, which could suggest a transient increase in ICP. However, after one month of continuous use, there was no significant change in ONSD compared to baseline, suggesting that the initial increase might be a short-term response rather than a sustained effect. Additionally, ONSD values did not show significant changes when the appliance was removed. This is the first study to demonstrate an increase in ICP in adolescents undergoing functional orthopedic treatment.

The lack of significant changes in ONSD after appliance removal (T4) suggests that the effect of the Twin Block appliance on ONSD is reversible and does not persist beyond the initial period of use. This finding is important for understanding the potential implications of using such appliances in patients with underlying conditions that might affect ICP.

A study on laryngoscopy involving the use of mouth gags found that when the laryngeal blade was positioned during suspension direct laryngoscopy, there was a significant increase in ONSD. This increase in ONSD persisted as long as the mouth gag remained in place [12]. A similar study involving the use of a mouth gag during adenotonsillectomy found a significant increase in ONSD. This increase in ONSD continued to rise as long as the mouth gag remained in place [11]. In another study, ONSD was examined by ultrasonography in a patient group who underwent fiberoptic bronchoscopy and it was observed that ONSD increased during bronchoscopy [18]. In all of the studies, the use of mouth gags or other tools caused the mandible to be depressed and shifted to a more inferior position, resulting in increased mouth opening. The vertical and sagittal positions of the mandible are also altered with the use of Twin Block appliances. Consistent with these changes, our study observed an increase in ONSD with the use of Twin Block.

Wardly et al. reported that in a patient who underwent maxillomandibular advancement and counterclockwise rotation surgery, jugular venous resistance decreased and cerebrospinal fluid absorption increased, thus improving ICP [19]. In Twin Block, although the mandible was moved to a more anterior position, an increase in ONSD was observed in the early period. This situation was thought to be due to increased tension in the perioral muscles and the adaptation mechanism not yet being formed. With long-term use, this adaptation developed and no difference was observed in ONSD, and data similar to this study were obtained.

Researchers have identified a correlation between ICP and ONSD in children. Specifically, when the ONSD exceeds 5.5 mm, ICP is often elevated by more than 20 mm Hg [4, 11, 20]. This is clinically important because an ICP increase greater than 15 mm Hg is classified as intracranial hypertension, while an elevation beyond 20 mm Hg is a critical threshold for treatment in cases of traumatic brain injury [21]. In this study, we found that ONSD increased significantly 1 min after the use of Twin Block.

The study’s findings have several clinical implications. The transient increase in ONSD observed shortly after the appliance was placed may be relevant for orthodontists to consider, particularly in patients with pre-existing conditions that could predispose them to elevated ICP. Although the long-term impact appears minimal, the initial rise in ONSD highlights the need for careful monitoring of patients who may be more susceptible to fluctuations in ICP.

The lack of significant changes in mean arterial pressure (MAP) after 10 min of appliance use and the absence of variations in SpO2 and heart rate suggest that the observed ONSD changes are not likely due to systemic cardiovascular changes.

In adults, mandibular advancement devices are acknowledged as an effective, non-invasive, and safe method for treating mild to moderate obstructive sleep apnea [22, 23]. However, this study focused on the impact of mandibular advancement on intracranial pressure in children, whose growth and development are still ongoing. More extensive research is required to explore the effects of mandibular advancement devices used for obstructive sleep apnea in adult patients on intracranial pressure.

Several limitations should be acknowledged in this study. The sample size was relatively small, with only 20 patients included in the study. A larger sample size would provide more robust results and enhance the generalizability of the findings. The study was conducted over a relatively short duration of one month, which may not fully capture the long-term effects of Twin Block appliance use on intracranial pressure or optic nerve sheath diameter. Longer follow-up periods could provide a clearer understanding of any sustained changes in ICP over time. The lack of control over the daily compliance of patients with appliance use could introduce variability, as it was self-reported by the patients and their families. In addition, while the use of ultrasonography to measure ONSD is a non-invasive and reliable method, it is not without limitations. Invasive techniques, like the insertion of intraparenchymal microtransducers or intraventricular catheters, are considered the gold standard for monitoring intracranial pressure. However, these methods carry the risk of complications, including bleeding and infection, which limits their practical application in clinical settings. While we used a non-invasive method to assess ICP, this approach may not offer the same level of precision as these invasive techniques, potentially affecting the accuracy of our measurements. The accuracy of ONSD measurements can be influenced by factors such as patient positioning, ultrasound technique, and the experience of the operator. Additionally, the optic nerve sheath may not always be perfectly circular. Finally, while this study focused on children with mandibular retrognathia, the findings may not be directly applicable to adult populations or those with different medical conditions that could affect ICP. Further research with more diverse populations and longer study durations is necessary to validate these results and explore the broader implications of mandibular advancement devices on ICP.

## Conclusion

This study demonstrates an increase in ICP in adolescents undergoing functional orthopedic treatment with the Twin Block appliance. Our findings suggest that the use of the Twin Block leads to a transient increase in ONSD, which may reflect a corresponding rise in ICP. This increase is most noticeable within the first minute of appliance use, with levels stabilizing over time and no significant long-term changes observed. These results highlight the importance of monitoring ONSD in patients undergoing orthodontic treatment with functional appliances, especially in those who may be at risk for elevated ICP.

## Electronic supplementary material

Below is the link to the electronic supplementary material.

Supplementary Material 1

Supplementary Material 2

## Data Availability

No datasets were generated or analysed during the current study.
